# Canakinumab therapy in patients with Familial Mediterranean Fever

**DOI:** 10.1186/1546-0096-13-S1-O45

**Published:** 2015-09-28

**Authors:** S Ugurlu, E Seyahi, G Hatemi, A Hacioglu, H Ozcan, FN Akkoc, H Ozdogan

**Affiliations:** 1 Cerrahpasa Medical Faculty, University of Istanbul, Division of Rheumatology, Department of Internal Medicine, Istanbul, Turkey

## Background

It has been reported that canakinumab, a monoclonal anti IL-1 antagonist, was effective in patients with FMF who are either non-responsive or intolerant to colchicine.

## Objectives

To share our experience with canakinumab in a larger group of FMF patients treated for diverse indications and with a longer follow-up.

## Methods

The data of the patients on canakinumab who are examined physically and checked for laboratory parameters before each injection is evaluated with regard to response and safety.

## Results

Data of 31 (17F/14M) patients who had received more than 3 injections of canakinumab (150mg/mo) were included. The indications were insufficient response to colchicine in 22 (>1 attack/month), amyloidosis in 6, injection site reaction with anakinra in 5, and adverse effects of colchicine in 4 patients (azoospermia and neuropathy in one each, myopathy in 2). Six patients had concomitant diseases like ankylosing spondylitis and polyarteritis nodosa. The mean age of the patients was 35,90±13 years, the mean disease duration was 15±9,30 years, the mean injection number was 9,80±6 and the mean duration of canakinumab therapy was 16±9,26 months. Twenty four patients had no attacks after canakinumab. The attack frequency was reduced more than %50 in 5 patients, did not change in 2. In 6 cases with FMF amyloidosis, proteinuria decreased in 2 (from 15020 to 1098mg/dl; from 6135 to 300mg/dl), increased in the other 2 (from 1700 to 4700mg/dl; from 5001 to 7061 mg/dl) and was stable in the remaining 2 patients. Fourteen patients with myalgia and calf pain unresponsive to colchicine, improved significantly as the mean patient global assessment score decreased from 8,25 ± 2,48 to 2,06±2,63 (p

## Conclusion

Canakinumab is effective in controlling the febrile attacks and calf pain in FMF patients with inadequate response or intolerant to colchicine, yet its effect in amyloidosis is variable. It seems to have an acceptable safety profile. Further controlled studies are needed to better assess the safety and efficacy of canakinumab in FMF.

